# Anatomical variations in termination of the uncal vein and its clinical implications in cavernous sinus dural arteriovenous fistulas

**DOI:** 10.1007/s00234-014-1383-6

**Published:** 2014-05-31

**Authors:** Satomi Ide, Hiro Kiyosue, Shuichi Tanoue, Mika Okahara, Yoshiko Sagara, Yuzo Hori, Hiromu Mori

**Affiliations:** Department of Radiology, Oita University Hospital, 1-1 Idaigaoka, Hasama, Yufu City, Oita 879-5593 Japan

**Keywords:** Cerebral vein, Dural arteriovenous fistula, Cerebral angiography

## Abstract

**Introduction:**

The aim of the study was to investigate the variations in the uncal vein (UV) termination and its clinical implication in cavernous sinus dural arteriovenous fistulas (CSDAVFs).

**Methods:**

Biplane cerebral angiography in 80 patients (160 sides) with normal cerebral venous return (normal group) was reviewed with special interest in the termination of the UV. Frequency and types of uncal venous drainage from CSDAVFs in consecutive 26 patients were also analyzed.

**Results:**

In the normal group, the UV was identified in 118 sides (74 %). The UV terminated into cavernous sinus (CS) in 41 sides (34 %), the superficial middle cerebral vein (SMCV) in 58 sides (48 %), the laterocavernous sinus (LCS) in 15 sides (13 %), and the paracavernous sinus (PCS) in 4 sides (3 %). Cerebral venous blood via the UV draining into the CS directly (*n* = 41) or through the SMCV and/or the LCS (*n* = 45) was observed in 86 sides (54 %). Uncal venous drainage from CSDAVFs was found in 13 patients (50 %). The CSDAVFs drained directly into the UV in two patients, drained via LCS into the UV in two patients, and drained through the SMCV into the UV in the remaining nine patients. All cases were successfully treated by transvenous embolization with special attention given to uncal venous drainage.

**Conclusion:**

There are several variations in UV termination according to the embryological development of the primitive tentorial sinus and the deep telencephalic vein. Careful attention should be paid to uncal venous drainage for the treatment of CSDAVFs.

## Introduction

There are many types of drainage route for cavernous sinus dural arteriovenous fistulas (CSDAVFs), namely: anterior drainage through the ophthalmic veins, inferior drainage through the inferior petrosal sinus and the pterygoid plexus, posterior drainage through the superior petrosal sinus, medial drainage through the intercavernous sinus, lateral drainage through the superficial middle cerebral vein, and deep drainage through the prepontine bridging vein, and uncal venous drainage [1–3]. Recognition of deep venous drainage routes from CSDAVFs is important because CSDAVFs with deep venous drainage carry a risk of deep cerebral bleeding [4]. Furthermore, serious complications such as brain edema or hemorrhage can occur when the dural arteriovenous fistulas (DAVFs) remain with a small cerebral venous drainage after transvenous embolization [5]. The uncal vein (UV) is a small cerebral vein in deep cerebral venous group, which is known as the anastomotic channel between the cavernous sinus (CS) and the basal vein of Rosenthal [6–8]. However, variations in termination of the UV have not been well-recognized. In this study, we investigate the variations in UV termination and its clinical significance in CSDAVFs.

## Materials and methods

Carotid cerebral angiography in 80 patients (160 sides) with normal cerebral venous return (normal group) was retrospectively reviewed with special interest in the termination of the UV. The patients who had lesions affecting cerebral venous drainage were excluded from this study. There were 35 males and 45 females, with ages ranging from 13 to 83 years (mean age, 56.5 years). All patients underwent selective cerebral angiography, including both internal or common carotid angiography and vertebral angiography using biplane angiography equipment (Infinix Celeve-i INFX-8000 V, Toshiba Medical, Tokyo, Japan) at our hospital between January 2011 and August 2012. Two experienced neuroradiologists (S. T and H. K) evaluated the images carefully by focusing on the visualization of the UV and its terminations.

We also reviewed 26 consecutive cases of CSDAVFs which were treated between September 2003 and January 2014 at our institutions. There were 7 males and 19 females, with ages ranging from 52 to 85 years (mean age, 69 years). Three patients were presented with aggressive symptoms including cerebral hemorrhage (*n* = 2) and brain stem edema (*n* = 1). All of the other 23 patients showed ocular symptoms and/or tinnitus. The CSDAVFs were classified into the three types according to the Borden’s classification depending on the type of venous drainage [9]. There were 4 type I arteriovenous fistulas (AVFs), 17 type II AVFs, and 5 type III AVFs.

Selective angiography of bilateral external carotid arteries, internal carotid arteries, and vertebral arteries were reviewed with special interest in the presence of drainage routes through the UV from the CSDAVFs. All cases were treated by transvenous embolization with coils.

Each patient gave written informed consent before cerebral angiography and intervention. Institutional review board approval is not required for retrospective studies at our institution.

## Results

### Termination of the UV in normal hemodynamics

**Fig. 1:**
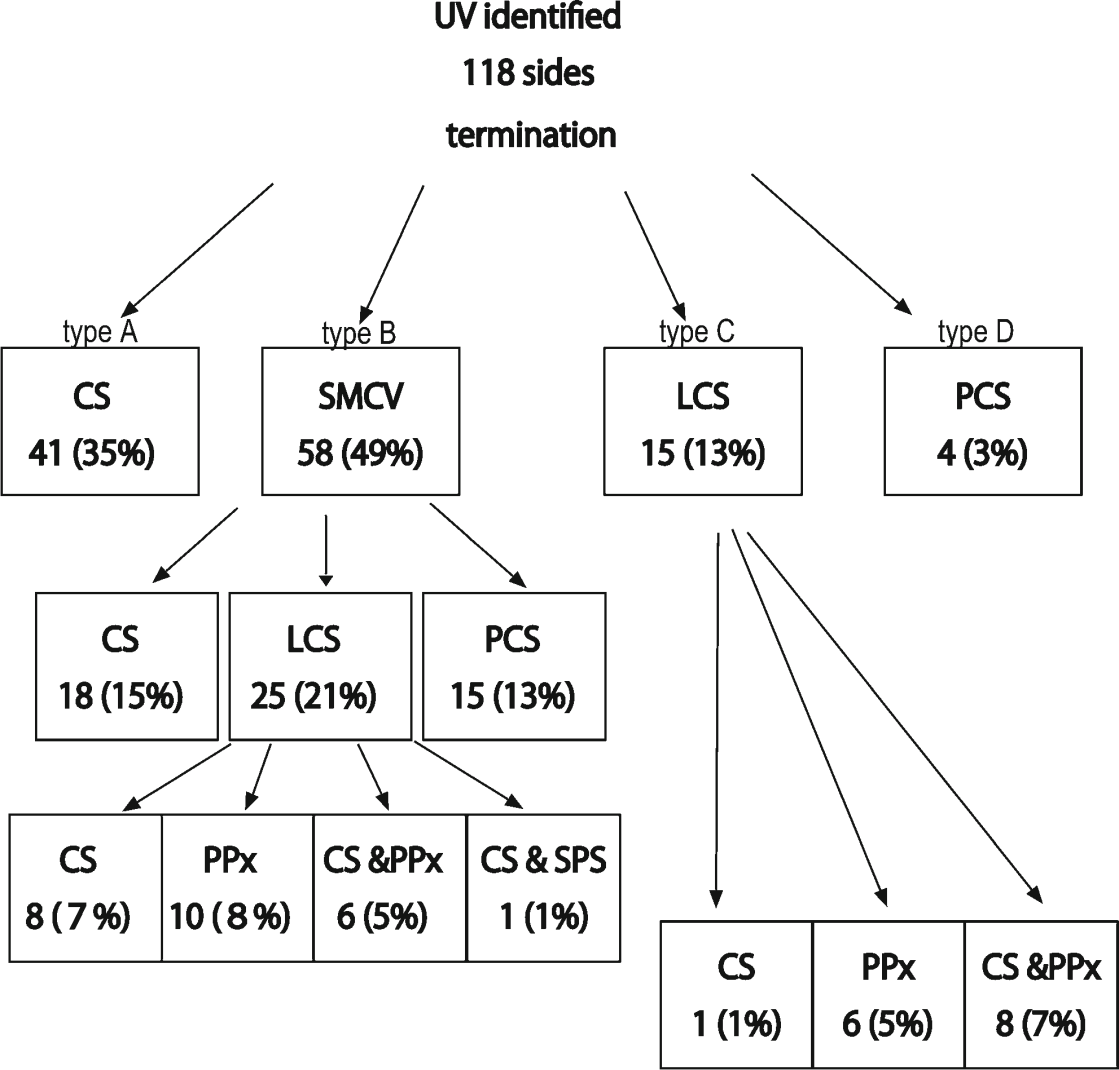
Diagram of terminations of the uncal vein in 118 patients with normal cerebral venous drainage

**Fig. 2:**
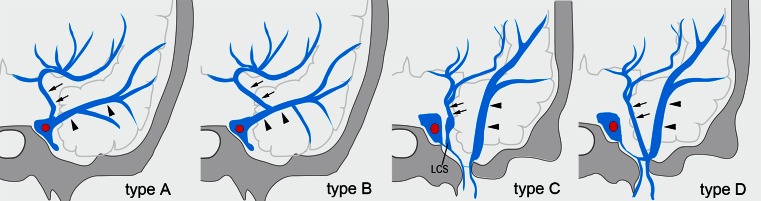
Types of termination of the uncal vein. *Type A*: The uncal vein terminated directly into the cavernous sinus (41 sides, 34 %). *Type B*: The uncal vein terminated into the superficial middle cerebral vein (58 sides, 48 %). *Type C*: The uncal vein terminated into the laterocavernous sinus (15 sides, 13 %). *Type D*: The uncal vein terminated into the paracavernous sinus (4 sides, 3 %). *Arrows* indicate the uncal vein, and arrowheads indicate the superficial middle cerebral vein

In the normal group, the UV was identified in 118 sides (74 %) of 160 sides. The UV terminated into the CS in 41 sides (type A, 35 %), the superficial middle cerebral vein (SMCV) in 58 sides (type B, 49 %), the laterocavernous sinus (LCS) in 15 sides (type C, 13 %), and the paracavernous sinus (PCS) in 4 sides (type D, 3 %) (Table 1; Figs. 1 and 2).

**Table 1:** Characteristics of 26 cases of CSDAVFs

Characteristics	No. of patients
Gender	
Male	7
Female	19
Symptoms	
Aggressive	3 (2 cerebral hemorrhage, 1 brain stem edema)
Non aggressive	23 (ocular symptoms with or without tinnitus)
Borden types	
Type 1	4
Type 2	17
Type 3	5
UV drainage	
Presence	13
Absence	13
Routes to the UV	
Directly from CS	2
via LCS	2
via SMCV	4
via LCS & SMCV	4
via PCS &SMCV	1
Other drainage routes	
SOV	19
SMCV	17
PPBV	9
IPS	8
SPS	8
ICS	6

*UV* uncal vein, *CS* cavernous sinus, *LCV* laterocavernous sinus, *SMCV* superficial middle cerebral vein, *PPBV* prepontine bridging vein, *IPS* inferior petrosal sinus, *SPS* superior petrosal sinus, *ICS* intercavernous sinus

**Fig. 3:**
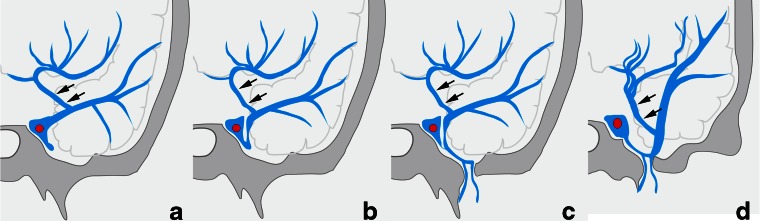
Schematic drawing of variation in termination of superficial middle cerebral vein in type B uncal venous termination. **a** The superficial middle cerebral vein (SMCV) terminated to the cavernous sinus. **b** The SMCV terminated to the laterocavernous sinus into the cavernous sinus. **c** The SMCV terminated to the laterocavernous sinus into the pterygoid plexus. **d** The SMCV terminated to the paracavernous sinus. *Arrows* indicate the uncal vein

In type B, the SMCV terminated into the CS in 18 sides, the LCS in 25 sides, and the PCS in 15 sides (Figs. 1 and 3). Among the 18 sides in which the SMCV terminated into the CS, the SMCV terminated into the CS alone in 16 sides, and the SMCV terminated into CS with another termination into the PCS in 2 sides. In the 25 sides where the SMCV terminated into the LCS, the LCS terminated into the CS in 8 sides, the pterygoid plexus in 6 sides, the CS and the pterygoid plexus in 10 sides, and the CS and the superior petrosal sinus in 1 side. The UV communicated with CS via the SMCV in 36 sides of type B.

In type C, the LCS terminated into the CS in one side, the pterygoid plexus in six sides, and the CS and the pterygoid plexus in eight sides. The UV communicated with CS via the LCS in nine sides of type C.

In type D, the PCS terminated into the pterygoid plexus in three sides and the transverse sinus in one side.

In the normal group, the deep cerebral venous blood via the UV draining into the CS directly (*n* = 41) or through the SMCV and/or the LCS (*n* = 45) was observed in 86 sides (54 %).

### Termination of the SMCV in each type of UV termination

**Fig. 4:**
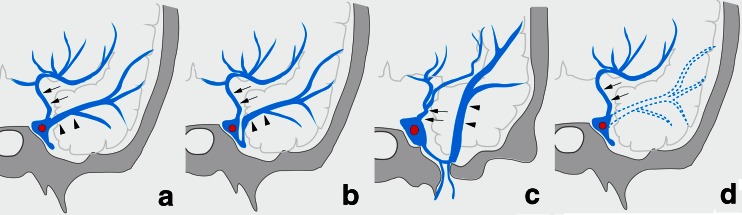
Schematic drawing of variation in termination of SMCV in type A uncal venous termination. **a** The uncal vein terminated directly to the cavernous sinus, and the SMCV terminated to the cavernous sinus. **b** The SMCV terminated to the laterocavernous sinus. **c** The SMCV terminated to the paracavernous sinus. **d** The SMCV is aplastic. *Arrows* indicate the uncal vein, and *arrowheads* indicate the superficial middle cerebral vein

**Fig. 5:**
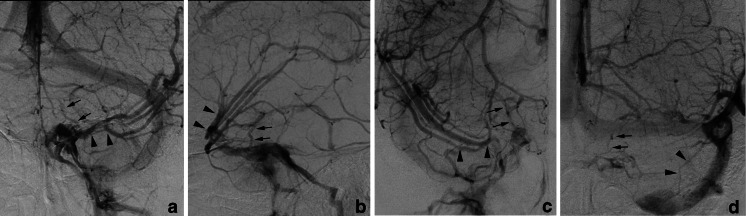
Type A uncal venous termination with various SMCV termination. **a** The SMCV terminating to the cavernous sinus. Left internal carotid angiography at venous phase shows the uncal vein (*arrows*) draining into the cavernous sinus and the SMCV (*arrowheads*) draining into the cavernous sinus anterolaterally. **b** The SMCV terminating to the laterocavernous sinus. Right internal carotid angiography at venous phase shows the uncal vein (*arrows*) draining into the cavernous sinus and the SMCV (*arrowheads*) draining through the laterocavernous sinus into the pterygoid plexus. **c** The SMCV terminating to the paracavernous sinus. Left internal carotid angiography at venous phase shows a small uncal vein (*arrows*) draining into the cavernous sinus and the small SMCV (*arrowheads*) draining into the paracavernous sinus

In type A, the SMCV terminated into the CS in 25 sides, the LCS in 4 sides, and the PCS in 7 sides (Figs. 4a–c and 5a–c). In five sides, the SMCV was not identified (aplastic) (Fig. 4d). Among the 25 sides in which the SMCV terminated into the CS, the SMCV terminated into the CS alone in the 22 sides. In the remaining three sides, the SMCV terminated into the CS with another termination into the PCS (*n* = 2) or the pharyngeal plexus (*n* = 1).

**Fig. 6:**
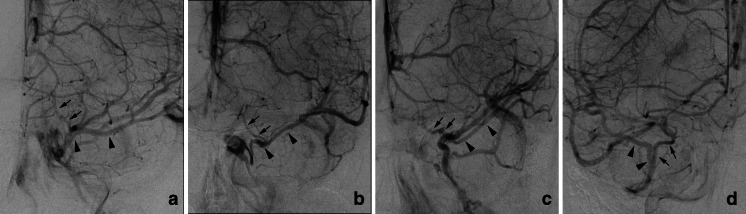
Type B uncal venous termination with various SMCV termination. **a** The SMCV terminating to the cavernous sinus. Left internal carotid angiography at venous phase shows the uncal vein (*arrows*) draining into the SMCV and the SMCV (*arrowheads*) draining into the cavernous sinus. **b** The SMCV terminating to the laterocavernous sinus into the cavernous sinus. Left internal carotid angiography at venous phase shows the uncal vein (*arrows*) draining into the SMCV and the SMCV (*arrowheads*) draining through the laterocavernous sinus into the cavernous sinus. **c** The SMCV terminating to the laterocavernous sinus into the pterygoid plexus. Left internal carotid angiography at venous phase shows the uncal vein (*arrows*) draining into the SMCV and the SMCV (*arrowheads*) draining through the laterocavernous sinus into the pterygoid plexus. **d** The SMCV terminating to the paracavernous sinus. Right internal carotid angiography at venous phase shows the uncal vein (*arrows*) draining into the SMCV and the SMCV (*arrowheads*) draining into the paracavernous sinus

In type B, the SMCV terminated into the CS in 18 sides (31 %), the LCS in 25 sides (43 %), and the PCS in 15 sides (26 %) as described before (Figs. 3 and 6).

**Fig. 7:**
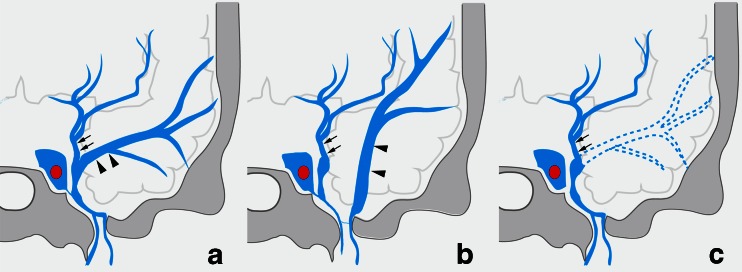
Schematic drawing of variation in termination of SMCV in type C uncal venous termination. **a** The uncal vein terminated to the laterocavernous sinus, and the SMCV terminated to the laterocavernous sinus. **b** The SMCV terminated to the paracavernous sinus. **c** The SMCV is aplastic

**Fig. 8:**
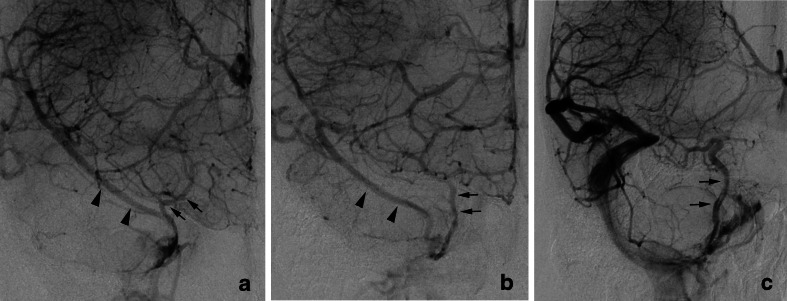
Type C uncal venous termination with various SMCV termination. **a** The SMCV terminating to the laterocavernous sinus. Right internal carotid angiography at venous phase shows the uncal vein (*arrows*) draining into the laterocavernous sinus and the SMCV (*arrowheads*) draining into the laterocavernous sinus separately. **b** The SMCV terminating to the paracavernous sinus. Right internal carotid angiography at venous phase shows the uncal vein (*arrows*) draining through the laterocavernous sinus to the pterygoid plexus and the SMCV (*arrowheads*) draining through the paracavernous sinus to the pterygoid plexus. **c** Aplastic SMCV. Right internal carotid angiography at venous phase shows a large uncal vein (*arrows*) draining through the laterocavernous sinus to the pterygoid plexus. The SMCV is not identified

In type C, the SMCV terminated into the LCS in 10 sides, and the PCS in 3 sides (Figs. 7a, b and 8a, b). The SMCV was absent in two sides (Figs. 7c and 8c). Among the 10 sides in which the SMCV terminated into the LCS, the LCS terminated into the CS alone in 2 sides, both the CS and the pterygoid plexus in 5 sides, and the pterygoid plexus alone in 3 sides.

**Fig. 9:**
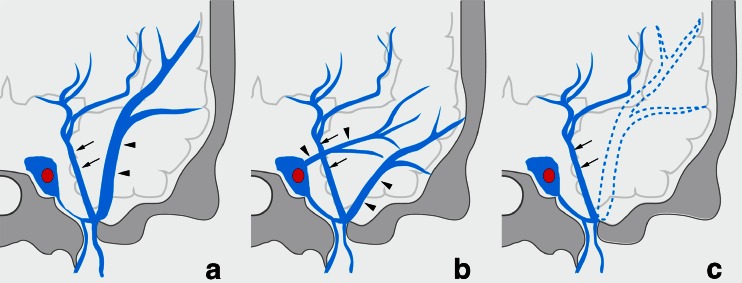
Schematic drawing of variation in termination of SMCV in type D uncal venous termination. **a** The uncal vein terminated to the paracavernous sinus, and the SMCV terminated to the paracavernous sinus. **b** The SMCV terminated to the paracavernous sinus and the cavernous sinus. **c** The SMCV is aplastic

**Fig. 10:**
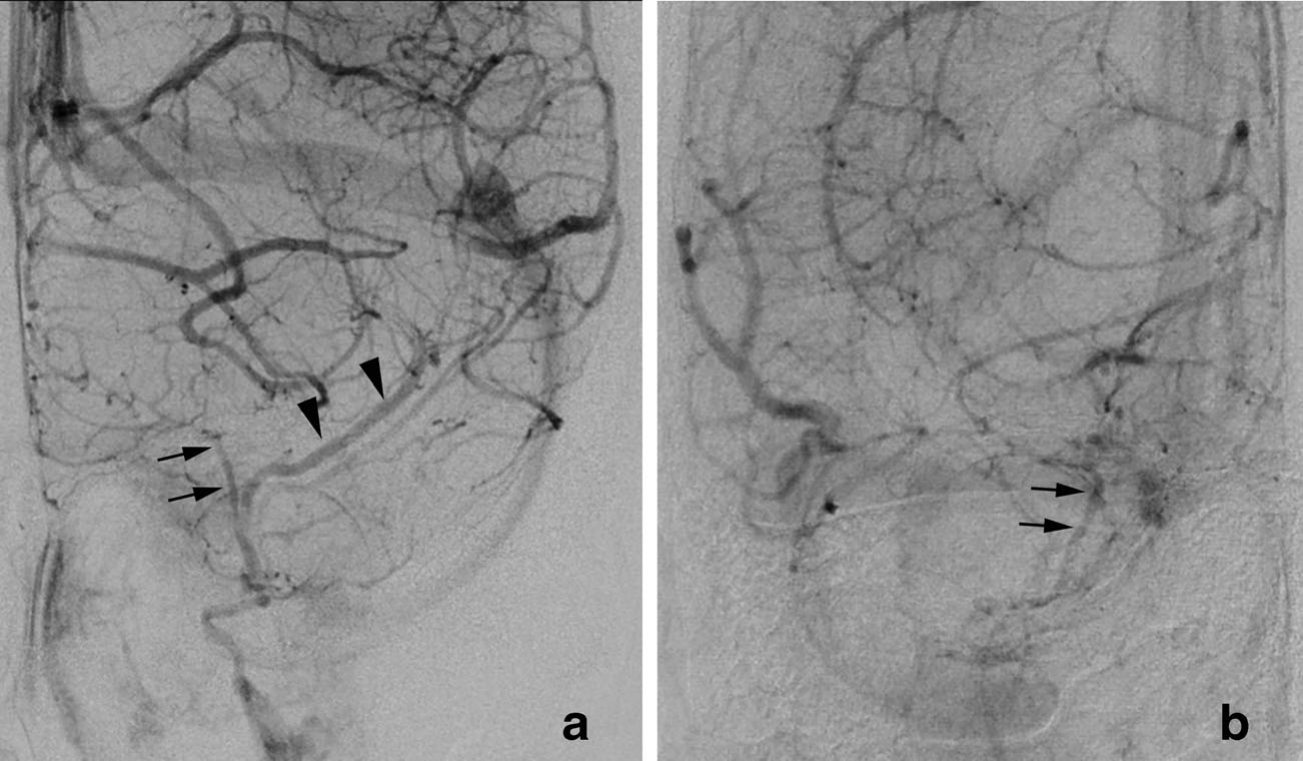
Type D uncal venous termination with various SMCV termination. **a** The SMCV terminating to the paracavernous sinus. Left internal carotid angiography at venous phase shows both the uncal vein (*arrows*) and the SMCV (*arrowheads*) draining into the paracavernous sinus separately. **b** Aplastic SMCV. Right internal carotid angiography at venous phase shows a the uncal vein (*arrows*) draining through the paracavernous sinus to the pterygoid plexus. The SMCV is not identified

In type D, the SMCV terminated into the PCS in two sides, and both CS and PCS in one side (Figs. 9 and 10). The SMCV was absent in one side.

### Cases of CSDAVFs

**Fig. 11:**
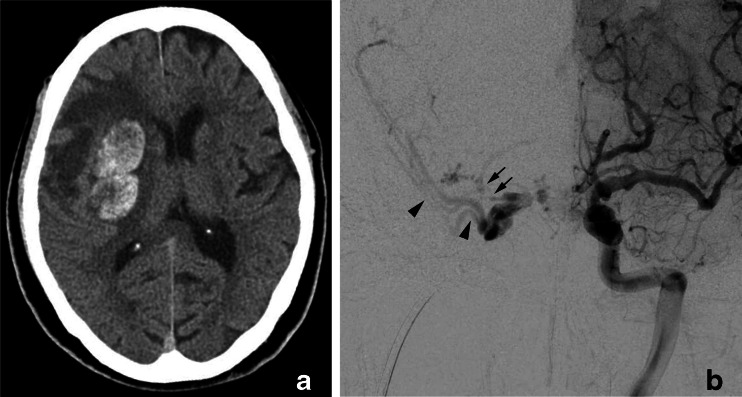
Cavernous sinus dural arteriovenous fistula with uncal venous drainage directly from the cavernous sinus. a CT shows right putaminal hemorrhage with perifocal edema. b Left internal carotid angiography shows the dural arteriovenous fistulas involving the right cavernous sinus. The AVFs drain into the superficial middle cerebral vein (*arrow heads*) and the uncal vein (*arrows*) directly from the right cavernous sinus

**Fig. 12:**
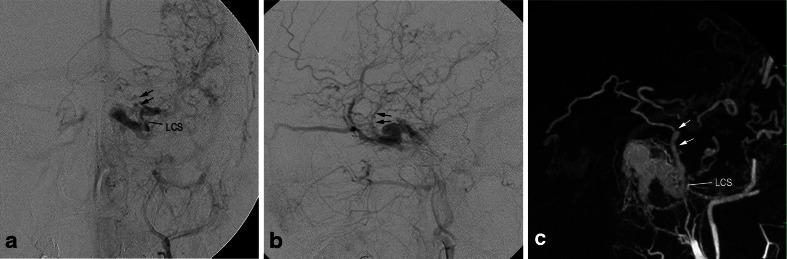
Cavernous sinus dural arteriovenous fistula with uncal venous drainage via the laterocavernous sinus. **a**, **b** Left external carotid angiography, frontal (**a**) and lateral (**b**) views show the dural arteriovenous fistulas involving the left cavernous sinus. The AVFs drain into the superficial middle cerebral vein and the superior ophthalmic vein. The uncal venous drainage (*arrows*) via the laterocavernous sinus (*LCS*) is also seen. **c** Coronal MIP image of the rotational angiography of the left external carotid artery clearly shows uncal venous drainage (*arrows*) via the laterocavernous sinus (*LCS*)

In the 26 cases of CSDAVFs, uncal venous drainage from CSDAVFs was found in 13 patients (50 %) (Table 1). CSDAVFs drained directly into the UV in two patients (Fig. 11), through the LCS into the UV in two patients (Fig. 12), through the SMCV into the UV in four patients, through the LCS and the SMCV to the UV in four patients, and through the paraCS and SMCV into the UV in one patient. All three patients presented with the aggressive symptoms showed Borden III type of the CSDAVFs. Among the three patients, the CSDAVFs drained retrogradely into the SMCV alone (cerebral hemorrhage), the SMCV and UV (cerebral hemorrhage) (Fig. 11), or the prepontine bridging vein (brain stem edema).

All cases were treated by transvenous embolization with special attention given to the small cortical venous drainage including uncal venous drainage, and the DAVFs disappeared without any complications.

## Discussion

The UV is a small cerebral vein located medially to the temporal lobe along the anterior margin of the uncus [6]. The peripheral tributes of the UV communicate with the first segment of the basal vein of Rosenthal, and the UV terminates into the SMCV or CS [7]. Therefore, the UV is known as a communicating channel between the CS and the basal vein of Rosenthal.

**Fig. 13:**
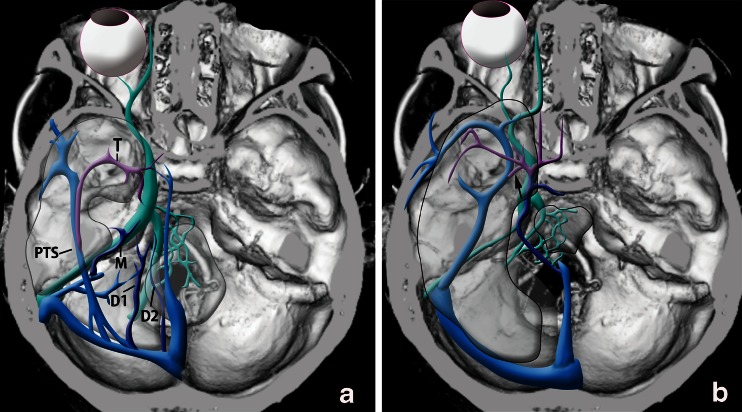
Schematic drawing of development of the UV. **a** early embryo. **D**uring the early embryonic stage, the deep telencephalic vein (*T*) flows into the primitive tentorial sinus (*PTS*) which is precursor of the superficial middle cerebral vein. The primitive tentorial sinus is displaced medially and connects with cavernous sinus according to the development of the temporal lobe. Later, the basal vein of Rosenthal is formed by anastomosis of the terminal branches of the deep telencephalic vein, the ventral (*D1*) and dorsal (*D2*) diencephalic vein, and the mesencephalic vein (*M*). **b** infant. **T**he uncal vein (*arrow*) consists of the remnant of the proximal part of the deep telencephalic vein

According to embryologic development of cerebral veins described by Padget, the UV derives from the deep telencephalic vein flowing into the primitive tentorial sinus which is the precursor of the superficial middle cerebral vein [10]. The primitive tentorial sinus runs posteriorly and connects to the transverse sinus. Later, the basal vein of Rosenthal is formed by the anastomosis of the terminal branches of the deep telencephalic vein, the ventral diencephalic vein, dorsal diencephalic vein, and mesencephalic vein (Fig. 13). Along with the growth of temporal lobes, the primitive tentorial sinus is displaced medially and connects with the CS. Several types in terminations of the superficial middle cerebral vein can occur depending on the degree of the connection between the primitive tentorial sinus and the CS. The several variations in UV termination as well as the SMCV can occur depending on the degree of development of these connections and the development of the basal vein of Rosenthal. However, only few papers have demonstrated the variations in UV termination [7, 8], and the types and frequency of these variations has not been well-known. In our results, the UV can be identified in 118 sides (74 %) of 160 sides on angiography in normal cerebral hemodynamic status. Among the 118 sides, the UV terminated into the CS in 35 %, the SMCV in 49 %, the LCS in 13 %, and the PCS in 3 %. The cerebral venous blood via the UV draining into the CS directly (*n* = 41) or through the SMCV and/or the LCS (*n* = 45), was observed in 86 sides (54 %). This anatomical variation in termination of the UV can be related to the UV drainage of the CSDAVFs which was found in 50 % of cases in this series. Cerebral hemorrhage of CSDAVFs is less than that of DAVFs at other locations because the CS connects with multiple emissary veins as well as the sinuses [3]. However, it was reported that the retrograde cortical venous drainage of CSDAVFs brings a high risk of intracerebral venous hemorrhage especially for cases with only a small cerebral venous drainage such as uncal vein [4]. In our series, aggressive behavior of CSDAVFs was seen in three cases (11.5 %). All three cases showed Borden type III AVF, and the UV drainage is related to the cerebral hemorrhage in one of the three cases. The aggressive symptoms would be more related to types of drainage such as Borden’s type III rather than presence of uncal venous drainage. However, cortical reflux into the small cerebral vein such as the uncal vein in type III AVF would have higher risk of aggressive symptoms.

CSDAVFs are generally treated by transvenous embolization. Careful attention should be paid to small cortical venous drainage such as uncal venous drainage during embolization because serious complications can occur when these retrograde drainage routes remain after transvenous embolization [5]. It is also important to know the variations in UV termination in cases of DAVFs for transvenous catheterization to the uncal venous drainage. Based on the angiographic study of LCS by Gailloud et al., LCS terminated into the CS at the posterior aspect in 32 %, and multiple terminations in 23 % [11]. Furthermore, anastomotic channels of small caliber linking the CS with LCS were observed in 36 %. Access route to the uncal venous drainage from the cavernous sinus is usually complex in a case which the uncal vein terminating into the LCS. Therefore, transvenous catheterization to the uncal venous drainage for cases where the UV joins to the CS via the LCS would be more difficult than other types of UV termination. Serious complication such as cerebral hemorrhage can occur when the uncal venous drainage remained solely as a drainage route of AVF after transvenous embolization.

In conclusion, there are several variations in UV termination, and this can be related to the uncal venous drainage of the CSDAVFs. It is important to recognize uncal venous drainage and the variations in UV termination for the treatment of CSDAVFs via a transvenous approach.
